# Health-related quality of life in adults after pediatric kidney failure in Switzerland

**DOI:** 10.1007/s00467-022-05760-6

**Published:** 2022-10-13

**Authors:** Marc-Andrea Heinzelmann, Claudia E. Kuehni, Katharina Roser, Luzius Mader, Guido F. Laube, on behalf of the Swiss Pediatric Renal Registry (SPRR) group

**Affiliations:** 1 Swiss Pediatric Renal Registry, Child and Adolescent Health Research Group, Institute of Social and Preventive Medicine, University of Bern, Bern, Switzerland; 2 Department of Pediatrics, University Children’s Hospital Bern, Bern, Switzerland; 3 Department of Health Sciences and Medicine, University of Lucerne, Lucerne, Switzerland; 4 Institute of Social and Preventive Medicine, University of Bern, Bern, Switzerland; 5 Department of Pediatrics, Hospital Baden, Baden, Switzerland

**Keywords:** Health-related quality of life, Pediatric kidney failure, Kidney replacement therapy, Short-Form 36

## Abstract

**Background:**

Little is known about health-related quality of life (HRQoL) in adults after kidney failure during childhood. In this study, we analyzed HRQoL of adults after pediatric kidney failure in Switzerland and investigated socio-demographic and clinical factors associated with HRQoL.

**Methods:**

In this cohort study, we sent questionnaires to 143 eligible patients registered in the Swiss Pediatric Renal Registry with continuous kidney replacement therapy starting before the age of 18 years. We assessed HRQoL using the Short-Form 36 version 1, compared HRQoL scores between our sample and the Swiss general population, and used linear regression models to examine socio-demographic and clinical factors associated with HRQoL.

**Results:**

We included 79 patients (response rate 55%) with a mean age of 38.6 years (range 19.4–63.1). Compared to the general population, HRQoL scores were lower for physical functioning (− 12.43, *p* < 0.001), role physical (− 13.85, *p* = 0.001), general health (− 14.42, *p* < 0.001), vitality (− 4.98, *p* = 0.035), and physical HRQoL (− 6.11, *p* < 0.001), but we found no difference in mental HRQoL (− 0.13, *p* = 0.932). The socio-demographic factors—lower education, unemployment, and not being in a relationship—were associated with lower HRQoL. The only clinical factor associated with HRQoL was the type of kidney disease. Patients with acquired kidney diseases had lower mental HRQoL than patients with congenital anomalies of the kidney and urinary tract (− 11.4, *p* = 0.007) or monogenetic hereditary diseases (− 9.5, *p* = 0.018).

**Conclusions:**

Adults after pediatric kidney failure in Switzerland have lower physical, but similar mental HRQoL compared to the general population. Subgroups may require special attention with regard to their HRQoL.

**Graphical abstract:**

**Figure:**
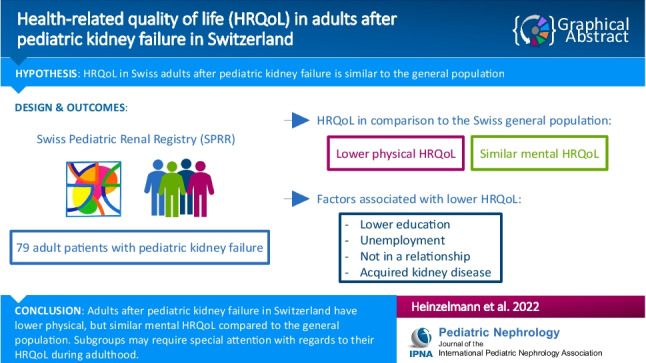
A higher resolution version of the Graphical abstract is available as Supplementary information

**Supplementary Information:**

The online version contains supplementary material available at 10.1007/s00467-022-05760-6.

## Introduction

Chronic kidney disease followed by kidney failure (KF) is rare in childhood. It is treated by kidney replacement therapy (KRT), including kidney transplantation, hemodialysis, or peritoneal dialysis. Patient and graft survival after pediatric kidney transplantation have steadily improved over the last decades [1, 2]. KF during childhood is associated with various long-term complications, such as cardiovascular disease, secondary malignancies, growth retardation, or side effects of long-lasting treatments (e.g., infections after immunosuppression) [3]. Psycho-social outcomes such as health-related quality of life (HRQoL), educational achievement, employment situation, civil status, and living arrangements can also be affected. In patients with adult-onset KF, HRQoL has become a crucial outcome parameter and lower HRQoL has been shown to be associated with a higher risk of death and hospitalizations [4].

Children after kidney transplantation reported HRQoL comparable to healthy children, although the parents indicated impaired HRQoL for their children [5]. HRQoL in adults after pediatric KF has rarely been examined. Studies that did not use standardized questionnaire instruments reported a good or even excellent quality of life [6–8]. However, HRQoL was rated poorer in comparison to the general population when standardized questionnaire instruments were used [9–12]. In this study, we aimed to describe HRQoL of adults who started receiving continuous KRT before the age of 18 years in Switzerland and investigate socio-demographic and clinical factors associated with HRQoL.

## Methods

### Study population

Patients were recruited from the Swiss Pediatric Renal Registry (SPRR), which had been established during the 1970s to gather data about children with KF in Switzerland [13]. We defined the following inclusion criteria for our study: (1) continuous KRT before the age of 18 years (kidney transplantation before the age of 18 years or start of dialysis before the age of 18 years followed by a kidney transplantation after the age of 18 years), (2) aged ≥ 18 years at study, (3) alive, and (4) formerly treated in a German-speaking children’s hospital in Switzerland. We decided to focus on the language region with the largest number of patients, for feasibility reasons in this unfunded study.

### Study design

This was a cross-sectional study nested in the longitudinal SPRR. We developed a questionnaire to assess a range of long-term outcomes in adults after pediatric KF with subsequent continuous KRT. The questionnaire was based on one we had used in a pilot study and the questionnaire of the Swiss Childhood Cancer Survivor Study [14], which assessed similar long-term outcomes. The questionnaire included items about the kidney disease, undergone treatments, comorbidities, current medications, education, profession, living arrangement, and lifestyle. We also included the Short-Form 36 (SF-36) version 1 as a standardized instrument for the evaluation of HRQoL [15]. We updated postal addresses of eligible patients via the national postal service and by contacting the hospitals the patients were transferred to after leaving pediatric care. Eligible patients had the option to either complete the questionnaire online or on paper. We sent two reminder letters with an additional copy of the questionnaire to all non-responders 1 and 2 months after initial contact, respectively.

### Evaluation of health-related quality of life

HRQoL was evaluated with the SF-36. It is one of the most extensively used questionnaire instruments to evaluate HRQoL and includes 36 items [16]. The SF-36 uses 35 out of 36 items to generate eight different subscales. Each item is exclusive to one subscale. The following eight subscales are defined: physical functioning (PF, 10 items), role physical (RP, 4 items), bodily pain (BP, 2 items), general health (GH, 5 items), vitality (VT, 4 items), social functioning (SF, 2 items), role emotional (RE, 3 items), and mental health (MH, 5 items). Higher subscale scores are indicative of a better health state (e.g. high scores for the subscale bodily pain imply absence of bodily pain) [15, 17]. The two summary scales, physical component summary (PCS) and mental component summary (MCS), are norm-based and weighted sums of the eight subscale scores [18]. PCS represents self-reported physical HRQoL and MCS self-reported mental HRQoL. Higher PCS and MCS scores represent better physical and mental HRQoL, respectively. The four subscales PF, RP, BP, and GH correlate primarily with PCS, whereas VT, SF, RE, and MH correlate mainly with MCS [18]. We compared our SF-36 version 1 scores to normative data from the general population of Switzerland for the SF-36 version 2 that were recently published [16]. Since standardized scoring is used, a direct comparison of results is possible [19].

### Socio-demographic and clinical variables

We assessed the following socio-demographic variables: age (continuous), gender (male, female), education (compulsory schooling or vocational training, upper secondary or university education) [20], employment status (employed or studying, unemployed), relationship status (in a relationship, not in a relationship), and living arrangement (living alone, not living alone).

Additionally, we investigated the following clinical variables obtained from the SPRR: type of kidney disease categorized into (1) congenital anomalies of the kidney and urinary tract (CAKUT), (2) monogenetic hereditary diseases, and (3) acquired diseases [13], age at first KRT (< 10 years, ≥ 10 years), and duration of KRT (< 25 years, ≥ 25 years). We assessed the type of KRT at time of study (dialysis, transplantation) and the number of transplants (1 transplant, > 1 transplant) in the questionnaire.

### Statistical analysis

First, we described HRQoL in our sample. The data collected from the questionnaires were evaluated according to the manuals for the SF-36 [15, 18]. Raw subscale scores are the sum of the items from the respective subscale. Missing raw subscale scores were calculated using the mean value, if at least half of the items for the respective scale were completed [15]. After raw subscale scores were calculated, they were transformed to a 0 to 100 scale, the so-called transformed scores. We used means and standard deviations from the Swiss general population as well as the transformed scores to generate a standardized score of every subscale. Then, we summed up standardized subscale scores using the corresponding factor score coefficients as weights to generate the PCS and MCS [16, 18]. Second, we compared the eight transformed subscale scores, PCS and MCS, to normative data from the Swiss general population using linear regression models adjusted for age and gender [16]. Third, we used linear regression models adjusted for age and gender to investigate socio-demographic and clinical variables associated with subscale scores, PCS and MCS. We performed all statistical analyses using Stata 16 (StataCorp LP, College Station, TX).

## Results

### Participants and their characteristics

**Fig. 1:**
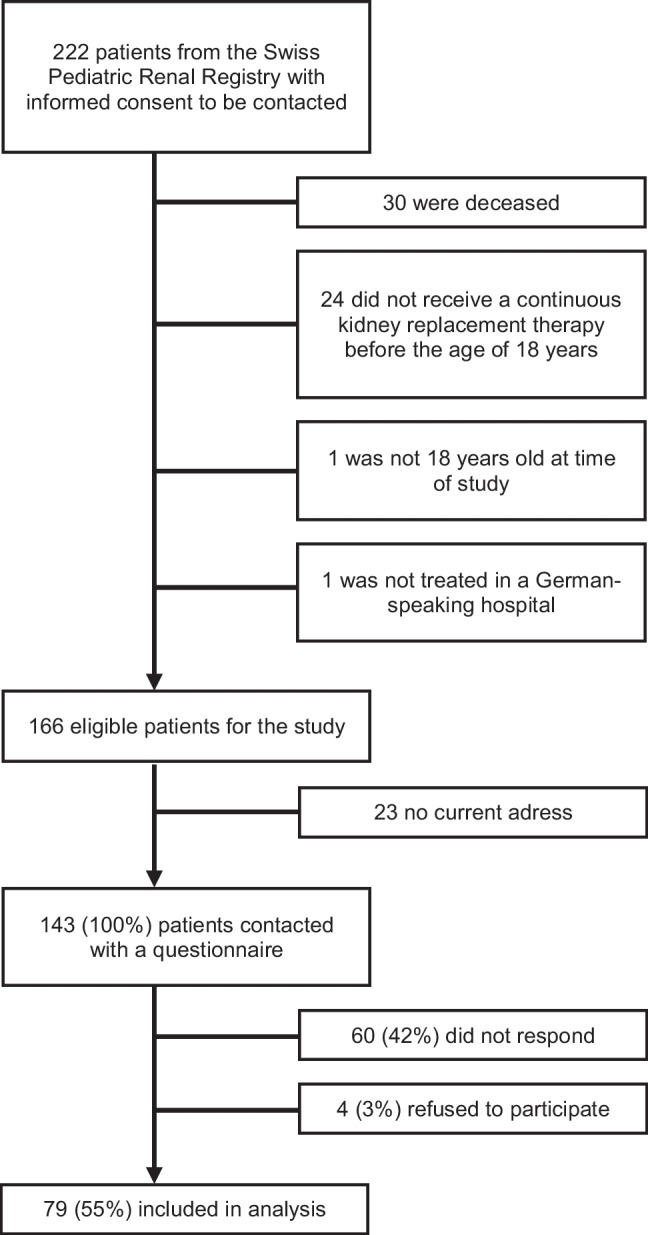
Flowchart of the study population

Out of 166 eligible patients for the study, we contacted 143 with the questionnaire (Fig. 1). Of those, 79 (55%) completed the questionnaire and were included in the analysis. One-third (34%) completed the survey online and 66% on paper. Socio-demographic and clinical characteristics of the participants are shown in Table 1. Mean age at study was 38.6 years (range: 19.4–63.1). Slightly more than half of the participants were male (57%). Almost half of participants (44%) had achieved upper secondary or university education. More than two-thirds of patients were employed or studying (76%) and 46% reported to be in a relationship. All patients had been transplanted. While a small number of patients (11%) had up to three kidney transplants and one-third (33%) had two transplants, most patients (56%) have had one transplant. The majority of participants (83%) reported having a functioning transplant at time of study. No differences were found between participants and non-participants for age, gender, type of kidney disease, age at start of KRT, and mean duration of KRT (all *p* > 0.05) (Online Resource 1).

**Table 1:** Characteristics of the participants

	(*n* = 79)	% of total
Mean age at time of study in years (range)	38.6 (19.4–63.1)	
Gender		
Female	34	43%
Male	45	57%
Education		
Compulsory schooling or vocational training	41	52%
Upper secondary or university education	35	44%
Missing	3	4%
Employment status		
Employed or studying	60	76%
Unemployed	19	24%
Relationship status		
In a relationship	36	46%
Not in a relationship	42	53%
Missing	1	1%
Living arrangement		
Living alone	26	33%
Not living alone	53	67%
Type of kidney disease		
Congenital anomalies of the kidney and urinary tract	29	37%
Monogenetic hereditary diseases	34	43%
Acquired diseases	16	20%
Age at first KRT		
Mean age at first KRT in years (range)	10.2 (0.0–17.9)	
< 10 years	32	41%
≥ 10 years	47	59%
Duration of KRT		
Mean duration of KRT in years (range)	28.4 (10.5–48.7)	
< 25 years	31	39%
≥ 25 years	48	61%
Type of KRT at time of study		
Transplantation	66	83%
Dialysis	10	13%
Missing	3	4%
Number of transplants		
1 transplant	44	56%
2 transplants	26	33%
3 transplants	9	11%

Abbreviations: *KRT* kidney replacement therapy

### SF-36 scores and comparison to the Swiss general population

Mean SF-36 subscale scores, PCS and MCS, of our study population and the Swiss general population are presented in Table 2. After adjusting for age and gender, we observed lower scores in the four subscales physical functioning (− 12.43, *p* < 0.001), role physical (− 13.85, *p* = 0.001), general health (− 14.42, *p* < 0.001), and vitality (− 4.98, *p* = 0.035) in our study population compared to the Swiss general population. No significant differences were found for the subscales bodily pain, social functioning, role emotional, or mental health (all *p* > 0.05). Our study population also showed a significantly lower PCS (− 6.11, *p* < 0.001), whereas we found no difference in MCS in comparison to the Swiss general population (− 0.13, *p* = 0.932).

**Table 2:** Mean SF-36 scores of the study population compared to the Swiss general population

	Study population mean (SD) (*n* = 79)	Swiss general population mean (SD) (*n* = 1209)	Adjusted difference^a^	*p* value
SF-36 subscale scores (0–100)				
Physical functioning (PF)	81.58 (25.29)	91.16 (17.01)	− 12.43	** < 0.001**
Role physical (RP)	75.66 (37.85)	86.41 (20.60)	− 13.85	**0.001**
Bodily pain (BP)	83.59 (24.81)	74.58 (26.03)	4.92	0.089
General health (GH)	62.98 (23.19)	75.64 (17.35)	− 14.42	** < 0.001**
Vitality (VT)	57.53 (20.53)	63.24 (17.22)	− 4.98	**0.035**
Social functioning (SF)	86.35 (20.43)	85.84 (20.02)	0.77	0.752
Role emotional (RE)	82.89 (34.21)	87.64 (19.22)	− 4.53	0.252
Mental health (MH)	74.91 (19.00)	75.02 (16.18)	0.61	0.781
SF-36 summary scales				
Physical component summary (PCS)	46.19 (14.02)	50 (10)	− 6.11	** < 0.001**
Mental component summary (MCS)	48.76 (12.67)	50 (10)	− 0.13	0.932

Abbreviations: SD, standard deviation; SF-36, Short-Form 36

^a^Adjusted for age and gender

### Associations between socio-demographic and clinical variables and SF-36 scores

Associations between socio-demographic and clinical variables and SF-36 scores are displayed in Tables 3 and 4. We found no association between age and gender with any of the SF-36 scores. Participants with compulsory schooling or vocational training scored lower on the subscales physical functioning (− 14.3, *p* = 0.007), role physical (− 17.4, *p* = 0.049), and vitality (− 11.9, *p* = 0.011) compared to those with upper secondary or university education. Unemployed participants scored lower on the subscales physical functioning (− 28.3, *p* < 0.001), role physical (− 26.9, *p* = 0.008), general health (− 19.9, *p* = 0.001), vitality (− 13.5, *p* = 0.012), and social functioning (− 19.6, *p* < 0.001) and had a lower PCS (− 12.9, p = 0.001) in comparison to participants who are employed or studying. Participants not in a relationship scored lower on the subscales physical functioning (− 17.7, *p* = 0.002) and general health (− 11.5, *p* = 0.031) and reported lower PCS (− 7.1, *p* = 0.029) compared to participants in a relationship. No associations between living arrangement and SF-36 scores were identified.

**Table 3:** Differences between subscale scores and component summary scores based on socio-demographic variables using linear regression models^a^

	Physical functioning (95% CI)	Role physical (95% CI)	Bodily pain (95% CI)	General health (95% CI)	Vitality (95% CI)	Social functioning (95% CI)	Role emotional (95% CI)	Mental health (95% CI)	Physical component summary (95% CI)	Mental component summary (95% CI)
**Age** (years)	− 0.5 (− 1.1, 0.1)	− 0.5 (− 1.4, 0.4)	− 0.4 (− 1.0, 0.2)	− 0.1 (− 0.6, 0.4)	0.0 (− 0.4, 0.5)	0.0 (− 0.5, 0.4)	0.0 (− 0.8, 0.8)	0.0 (− 0.4, 0.5)	− 0.3 (− 0.6, 0.1)	0.1 (–0.2, 0.4)
**Gender**										
Male (ref)										
Female	− 1.8 (− 13.3, 9.6)	− 4.1 (− 21.8, 13.6)	− 5.9 (− 17.4, 5.6)	− 3.6 (− 14.4, 7.2)	− 9.0 (− 18.4, 0.4)	− 3.3 (− 12.9, 6.3)	− 11.1 (− 27.1, 4.9)	− 7.0 (− 15.7, 1.8)	− 0.6 (− 7.2, 6.1)	− 4.5 (− 10.5, 1.5)
**Education**										
Higher^b^ (ref)										
Lower^c^	− 14.3* (− 24.7, − 4.0)	− 17.4* (− 34.7, − 0.1)	− 8.5 (− 20.2, 3.2)	− 8.9 (− 19.7, 1.9)	− 11.9* (− 21.0, − 2.8)	− 7.7 (− 17.6, 2.1)	− 10.1 (− 26.5, 6.2)	− 5.9 (− 14.8, 3.0)	− 6.1 (− 12.5, 0.2)	− 4.0 (− 10.1, 2.1)
**Employment status**										
Employed (ref)										
Unemployed	− 28.3** (− 40.0, − 16.7)	− 26.9* (− 46.7, − 7.1)	− 10.0 (− 23.0, 3.0)	− 19.9* (− 31,8, − 8.0)	− 13.5* (− 24.0, − 3.1)	− 19.6** (− 29.9, − 9.3)	− 17.0 (− 35.3, 1.2)	− 5.4 (− 15.5, 4.7)	− 12.9* (− 20.3, − 5.6)	− 5.8 (− 13.0, 1.2)
**Relationship status**										
In a relationship (ref)										
Not in a relationship	− 17.7* (− 28.5, − 7.0)	− 13.6 (− 31.1, 3.9)	− 1.1 (− 12.6, 10.5)	**–** 11.5* (− 22.0, − 1.1)	− 3.4 (− 12.7, 6.0)	− 2.9 (− 12.5, 6.6)	0.1 (− 16.0, 16.2)	− 3.3 (− 12.1, 5.4)	− 7.1* (− 13.5, − 0.8)	0.2 (− 5.8, 6.2)
**Living arrangement**										
Not living alone (ref)										
Living alone	1.5 (− 10.6, 13.7)	8.8 (− 9.8, 27.4)	4.7 (− 7.5, 16.9)	0.0 (− 11.6, 11.5)	2.1 (− 7.8, 12.1)	2.3 (− 7.9, 12.6)	14.4 (− 2.2, 31.1)	− 0.8 (− 10.1, 8.5)	2.4 (− 4.8, 9.5)	2.1 (− 4.4, 8.5)

Abbreviations: *CI*, confidence interval; *ref*, reference value

^a^Adjusted for age and gender

^b^Upper secondary or university education

^c^Compulsory schooling or vocational training

^*^p value < 0.05, **p value < 0.001

**Table 4:** Differences between subscale scores and component summary scores based on clinical variables using linear regression models^a^

	Physical functioning (95% CI)	Role physical (95% CI)	Bodily pain (95% CI)	General health (95% CI)	Vitality (95% CI)	Social functioning (95% CI)	Role emotional (95% CI)	Mental health (95% CI)	Physical component summary (95% CI)	Mental component summary (95% CI)
**Type of kidney disease**										
Acquired diseases (ref)										
CAKUT	3.6 (− 12.8, 20.0)	4.8 (− 20.2, 29.8)	4.5 (− 11.8, 20.8)	5.9 (− 9.6, 21.4)	9.7 (− 3.4, 22.8)	13.1 (− 0.5, 26.7)	28.3* (6.7, 49.9)	11.8 (− 0.4, 24.0)	− 1.0 (− 10.5, 8.5)	11.4* (3.2, 19.6)
Monogenetic hereditary diseases	3.1 (− 12.7, 18.9)	9.1 (− 15.0, 33.2)	6.7 (− 8.8, 22.2)	12.0 (− 3.0, 27.0)	11.7 (− 0.9, 24.2)	12.4 (− 0.6, 25.5)	23.2* (2.4, 43.9)	6.7 (− 5.0, 18.4)	3.4 (− 5.7, 12.4)	9.5* (1.7, 17.4)
**Age at first KRT**										
≥ 10 years (ref)										
< 10 years	− 6.8 (− 20.1, 6.6)	3.8 (− 16.7, 24.3)	4.8 (− 8.6, 18.2)	5.1 (− 7.5, 17.7)	1.6 (− 9.3, 12.5)	1.5 (− 9.7, 12.7)	4.6 (− 13.8, 22.9)	4.3 (− 5.9, 14.4)	− 1.2 (− 9.0, 6.6)	2.0 (− 5.0, 9.0)
**Duration of KRT**										
≥ 25 years (ref)										
< 25 years	− 2.4 (− 17.1, 12.2)	− 2.1 (− 24.5, 20.2)	− 8.4 (− 22.8, 5.9)	− 10.0 (− 23.6, 3.6)	− 2.4 (− 14.3, 9.5)	− 4.0 (− 16.1, 8.1)	− 4.0 (− 24.1, 16.0)	− 1.4 (− 12.6, 9.7)	− 3.4 (− 11.7, 4.9)	− 0.5 (− 8.0, 7.0)
**Type of KRT at study**										
Transplantation (ref)										
Dialysis	− 11.2 (− 28.6, 6.2)	− 12.4 (− 38.8, 14.1)	− 8.2 (− 25.5, 9.2)	− 10.2 (− 26.6, 6.3)	− 4.4 (− 18.9, 10.0)	− 4.2 (− 18.8, 10.3)	− 2.9 (− 26.6, 20.7)	0.2 (− 13.2, 13.5)	− 7.9 (− 17.5, 1.7)	0.3 (− 8.5, 9.0)
**Number of transplants**										
1 transplant (ref)										
> 1 transplant	− 6.6 (− 19.6, 6.4)	1.5 (− 18.9, 22.0)	0.6 (− 12.6, 13.9)	7.6 (− 4.8, 20.1)	1.7 (− 9.2, 12.5)	− 4.7 (− 15.8, 6.3)	− 8.1 (− 26.3, 10.1)	− 1.4 (− 11.6, 8.7)	1.5 (− 6.2, 9.1)	− 1.7 (− 8.6, 5.2)

Abbreviations: *CAKUT*, congenital anomalies of the kidney and urinary tract; *CI*, confidence interval; *KRT*, kidney replacement therapy; ref, reference value

^a^Adjusted for age and gender

^*^p value < 0.05

In terms of clinical characteristics, only the type of kidney disease was associated with SF-36 scores. We found that patients with CAKUT and monogenetic hereditary diseases scored significantly higher in the subscale role emotional (28.3, *p* = 0.011, and 23.2, *p* = 0.029, respectively) and showed a higher MCS (11.4, *p* = 0.007, and 9.5, *p* = 0.018, respectively) compared to patients with acquired kidney diseases. We found no associations between age at first KRT, duration of KRT, type of KRT at time of study, and number of transplants and SF-36 scores.

## Discussion

Our study shows that adults who have experienced pediatric KF have a lower physical but similar mental HRQoL compared to the general population. Lower education, unemployment and not being in a relationship were identified as the most important socio-demographic determinants of lower HRQoL. Type of kidney disease was the only clinical factor associated with HRQoL. Patients with acquired kidney diseases reported lower mental HRQoL than patients with CAKUT or monogenetic hereditary diseases.

For a better overview of the results and studies discussed in the following paragraphs, we provided a table in the supplementary material (Online resource 2). Regarding physical HRQoL our study population scored lower in the domains physical functioning, role physical, general health, and in the PCS, than the Swiss general population. A Norwegian study investigating young adults (mean age: 29.8 years) after kidney transplantation during childhood and early adulthood had obtained similar findings. Participants also had lower scores in all predominantly physical subscales of the SF-36, except bodily pain, and for the PCS overall [11]. A Dutch study including participants with a similar mean age (40.6 years) surveyed with the RAND-36 questionnaire, which is almost identical to the SF-36, reported similar results regarding the subscales mainly being associated with physical HRQoL. This population scored lower on the subscales physical functioning and general health, but higher in the subscale bodily pain in comparison to the general population [12]. Collectively, these findings suggest that physical HRQoL is impaired in adults after pediatric KF. This is also reflected in some comments made by our patients in the questionnaire indicating that one of their main daily life problems is physical fatigue.

One study has observed that adults after pediatric KF reported less pain in comparison to the general population [12]. Although not significant, we observed similar findings in our study. One explanation may be that transplanted patients have experienced poor health and strong pain in the past, which may have altered their level of pain tolerance. Additionally, these patients might be more familiar with adequate pain management, as they visit their healthcare provider several times a year.

While adults after pediatric KF in our study scored lower on the subscale vitality, MCS and the other subscales mainly being associated with mental HRQoL did not significantly differ from the Swiss general population. An explanation for the lower vitality score may be the diminished physical health, since vitality is a subscale that also has a notable correlation to the PCS [17]. These reassuring findings regarding mental health are consistent with other studies of adults after pediatric KF [9, 12]. Studies investigating adults after pediatric liver transplantation did not show lower scores for MCS or any other mainly mental SF-36 score when compared to the general population [21–23]. Also, a study of adults after heart transplantation during childhood found no difference in mental HRQoL when compared to the general population [24]. These findings suggest that patients requiring transplantation during childhood are mentally resilient, because they successfully cope with the burden of being chronically ill. It has also been speculated that patients with a chronic disease since childhood lack the experience of living without impairment and therefore life meets their expectations [12]. Overall, these studies indicate an excellent outcome regarding mental HRQoL in transplanted children who reached adult age, encouraging and justifying organ transplantation programs during childhood. Patients aged 12–25 on dialysis reported low HRQoL values [25], further indicating that kidney transplantation is the treatment of choice. 

Long-term physical and mental HRQoL outcomes in our study population seem to be very similar, or in the case of mental HRQoL even slightly better, compared to those of patients after kidney transplantation during adulthood. A systematic review investigating these patients concluded that physical HRQoL was impaired and mental HRQoL was “lower to comparable” when compared to the general population or healthy controls [26].

Another group of patients where long-term outcome is of great interest are survivors of childhood cancer. The physical and mental HRQoL of Swiss survivors of childhood cancer with a mean age of 25 years were both higher than what was reported in the general population, and the mental HRQoL of survivors was even better than that of their siblings [27]. In contrast to childhood cancer patients, the patients in our study population are not able to go into remission and need life-long immunosuppressive medication and intermittent dialysis treatment. Together with the younger age of the childhood-cancer cohort this may be an explanation for the observed differences.

We did not find associations between age or gender and HRQoL. These findings are in line with past studies with adults with childhood-onset KF [9, 12, 28]. We found that a higher education was associated with better HRQoL. This was also observed in the Swiss general population [16] and among patients with adult-onset KF [29]. Children with mild to moderate chronic kidney disease have been reported to function less well at school [30]. For children with kidney failure, we assume that this is also the case, if not worse, because of frequent hospital appointments for dialysis or follow-up visits after transplantation. Many of our participants left comments that these repeated visits to the hospital affected their performance in school. In Switzerland, there are some schooling programs available in pediatric hospitals, but they are not standardized and largely rely on individual commitments and funding of the hospital. Given that a higher educational achievement is associated with a better HRQoL, such programs could contribute to mitigate adverse outcomes in the long-term.

Our study further indicates that unemployment is a strong predictor of lower HRQoL. This was also found in the Swiss general population [16], patients with adult-onset KF [29] and adults with childhood-onset KF [12]. This is substantiated by free-text feedback from participants, emphasizing that they felt disadvantaged when it came to employment opportunities because of their kidney disease. We also found that participants in a relationship had a higher score in the subscales physical functioning, general health and the PCS. While being in a relationship itself can be invigorating, patients in a relationship may also have a better physical HRQoL because their partner can motivate and support them to be more physically active. It may also be that patients with a better physical health are more likely to find a partner. Overall, it seems that the socio-demographic characteristics such as education, employment status, or relationship status have a stronger correlation to the physical HRQoL than the mental HRQoL. Furthermore, the negative impact of being unemployed or single on HRQoL seems to be greater in the study population than the Swiss general population, especially when comparing the effects on physical HRQoL [16].

Our findings suggest that patients with acquired diseases (*n* = 16) have a worse mental HRQoL compared to patients with congenital anomalies of the kidney and urinary tract or monogenetic hereditary diseases. Patients with an acquired disease experienced a period in life without being chronically ill. It can only be speculated, whether this explains their impaired mental HRQoL during adulthood. Studies showed that patients with adult-onset KF had a better HRQoL after a kidney transplantation compared to patients on dialysis [26, 31]. In our study we did not find an association between treatment modality at time of study and HRQoL. Comparable findings were described in a previous study investigating adult patients with pediatric KF [12]. An explanation may be that after years of KRT since childhood, adult patients have accepted it as a part of themselves and are less influenced by their type of treatment. In accordance with previous studies, other clinical variables such as age at start of KRT, duration of KRT, or number of transplantations were not associated with HRQoL in adulthood [9, 12, 28].

The main limitation of our study is the relatively small sample size limiting statistical power for subgroup comparisons, especially when comparing modality of treatment at time of study. Although we found no differences when comparing participants and non-participants, our findings might be affected by selection or non-response bias, since patients with a better or worse HRQoL may have been more likely to complete the questionnaire. Additionally, our study lacked information on other potential risk factors for lower HRQoL in chronic kidney disease such as single parent household, anemia, hypertension and mood disorders [32].

In summary, adults after pediatric kidney failure in Switzerland have lower physical, but similar mental HRQoL compared to the general population. This study contributes to increase the awareness about long-term outcomes in adults after pediatric KF and highlights subgroups being at risk of impaired HRQoL during adulthood.

## Supplementary Information

Below is the link to the electronic supplementary material.

Graphical Abstract (PPTX 339 KB)

Supplementary file2 (DOCX 23 KB)

## Data Availability

The data that support the information of this manuscript were accessed on secured servers of the Institute of Social and Preventive Medicine at the University of Bern. Data can only be made available for researchers who fulfil the respective legal requirements. All data requests should be communicated to the corresponding author.
