# Supplementary material for: Health-related quality of life in adults after pediatric kidney failure in Switzerland
Source: Pediatr Nephrol. 2022 Oct 13;38(5):1559–68. doi: 10.1007/s00467-022-05760-6 (PMC10060264; doi:10.1007/s00467-022-05760-6)
Supplement: Supplementary file 1 — Graphical Abstract (PPTX 339 KB) [file 467_2022_5760_MOESM1_ESM.pptx]

## Slide 1
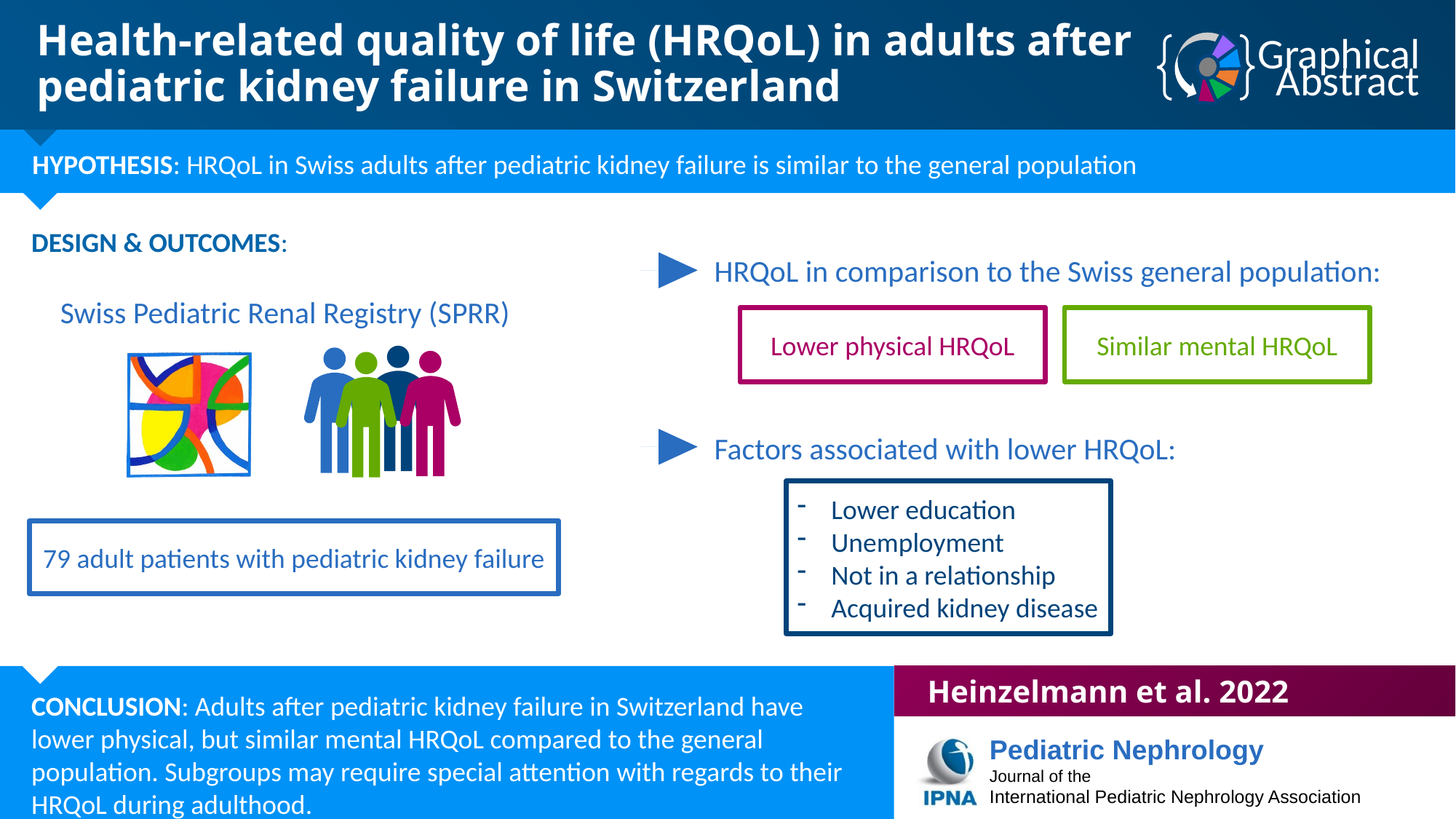

Health-related quality of life (HRQoL) in adults after
pediatric kidney failure in Switzerland
HYPOTHESIS: HRQoL in Swiss adults after pediatric kidney failure is similar to the general population
DESIGN & OUTCOMES:
HRQoL in comparison to the Swiss general population:
Swiss Pediatric Renal Registry (SPRR)
Similar mental HRQoL
Lower physical HRQoL
Factors associated with lower HRQoL:
Lower education
Unemployment
Not in a relationship
Acquired kidney disease
79 adult patients with pediatric kidney failure
Heinzelmann et al. 2022
CONCLUSION: Adults after pediatric kidney failure in Switzerland have lower physical, but similar mental HRQoL compared to the general population. Subgroups may require special attention with regards to their HRQoL during adulthood.
