# Supplementary material for: Health-related quality of life in adults after pediatric kidney failure in Switzerland
Source: Pediatr Nephrol. 2022 Oct 13;38(5):1559–68. doi: 10.1007/s00467-022-05760-6 (PMC10060264; doi:10.1007/s00467-022-05760-6)
Supplement: Supplementary file 2 — Supplementary file2 (DOCX 23 KB) [file 467_2022_5760_MOESM2_ESM.docx]

## Journal of Pediatric Nephrology

## Health-related quality of life in adults after pediatric kidney failure in Switzerland

## Authors and Affiliations

Marc-Andrea Heinzelmann^1^, Claudia E. Kuehni^1,2^, Katharina Roser^3^, Luzius Mader^4^*, Guido F. Laube^5^*, on behalf of the Swiss Pediatric Renal Registry (SPRR) group

^1^Swiss Pediatric Renal Registry, Child and Adolescent Health Research Group, Institute of Social and Preventive Medicine, University of Bern, Bern, Switzerland

^2^Department of Pediatrics, University Children's Hospital Bern, Bern, Switzerland

^3^Department of Health Sciences and Medicine, University of Lucerne, Lucerne, Switzerland

^4^Institute of Social and Preventive Medicine, University of Bern, Bern, Switzerland

^5^Department of Pediatrics, Hospital Baden, Baden, Switzerland

*These authors contributed equally to this work (shared last authorship).

### Corresponding author

Marc-Andrea Heinzelmann

Email: marc-andrea.heinzelmann@ispm.unibe.ch

| **Online Resource 1.** Comparison of participants and non-participants, based on information from the SPRR | | | |
| --- | --- | --- | --- |
|  | Participants  (n = 79) | Non-participants  (n = 87) | p-value |
| Mean age at time of study in years (SD) | 38.6 (10.1) | 40.2 (10.8) | 0.296 |
| Gender |  |  | 0.934 |
| Female | 34 (43%) | 38 (44%) |  |
| Male | 45 (57%) | 59 (56%) |  |
| Type of kidney disease |  |  | 0.952 |
| CAKUT | 29 (37%) | 34 (39%) |  |
| Monogenetic hereditary disease | 34 (43%) | 36 (41%) |  |
| Acquired disease | 16 (20%) | 17 (20%) |  |
| Mean age at start of KRT in years (SD) | 10.2 (5.4) | 11.3 (4.2) | 0.131 |
| Mean duration of KRT in years (SD) | 28.4 (9.0) | 28.9 (10.0) | 0.700 |
| Abbreviations: CAKUT, congenital anomalies of the kidney and urinary tract; KRT, kidney replacement therapy; SD, standard deviation; SPRR, Swiss Pediatric Renal Registry | | | |

| **Online Resource 2.** Overview of previous studies investigating HRQoL after pediatric transplantation | | | | |
| --- | --- | --- | --- | --- |
| Author (year) | Country | Study population | Instrument to assess HRQoL^a^ | Findings |
| Konidis (2015) | Canada | 27 participants, mean age 24 years, ≥ 15 years after pediatric liver transplantation | SF-36 version 2 | Patients that were older than 18 years at time of study (n=20), showed a lower score for the subscale general health only. |
| Tjaden (2014) | Netherlands | 89 participants, aged 32-52 years, started KRT at < 15 years old | RAND-36 | Overall, patients showed a lower score for the subscales physical functioning, vitality and general health. Factors associated with an impaired HRQoL included unemployment, comorbidities and disabilities. |
| Tozzi (2012) | Italy | 66 participants, aged 18-34 years, after pediatric kidney transplantation | SF-36 | Only a subset of patients who underwent a kidney transplant showed low HRQoL scores. The presence of a severe comorbidity was the only factor associated with lower PCS or MCS scores. |
| Mohammad (2012) | USA | 30 participants, aged 18-22 years, after pediatric liver transplantation | SF-36 version 2 | A clinically important difference was found in the subscale general health, for which the study population scored lower, and vitality, for which the study population scored higher. No difference in PCS or MCS was found in comparison to the control population. |
| Kosola (2012) | Finland | 57 participants, aged 3-35 years, after pediatric liver transplantation | SF-36 | Patients that were older than 18 years at time of study (n=29), showed lower scores for the subscales physical functioning and general health. Patients and controls scored similarly regarding emotional well-being. |
| Petroski (2009) | USA | 23 participants, aged 18-34 years, ≥ 10 years after pediatric heart transplantation | SF-36 version 2 | The mean scores of the cohort for all subscales were similar to those of the control population despite frequent late complications. |
| Aasebo (2009) | Norway | 131 adult participants, mean age 30 years, kidney transplantation between the age of 8-34 | SF-36 | In comparison to the general population the study population reported lower scores for all scales of the SF-36, except bodily pain. |
| Groothoff (2003) | Netherlands | 131 participants, mean age 29 years, started KRT < 15 years old | RAND-36 | Transplanted patients had lower scores for physical functioning, general health and role physical. Patients on dialysis scored lower in the subscales physical functioning, social functioning, role physical, general health and the PCS. Having a disability or comorbidity was identified as a determinant of lower HRQoL in transplanted patients. |
| Abbreviations: HRQoL, health-related quality of life; KRT, kidney replacement therapy; MCS, mental component summary; PCS, physical component summary, RAND-36, RAND-36 Health Survey; SF-36, Short-Form 36  ^a^Only instruments whose results are discussed in this study are listed | | | | |
